# Retrospective discrimination of PNES and epileptic seizure types using blood RNA signatures

**DOI:** 10.1007/s00415-024-12877-1

**Published:** 2025-01-15

**Authors:** Katie Bullinger, Monica Dhakar, Andrea Pearson, Argyle Bumanglag, Emine Guven, Rashi Verma, Elham Amini, Robert S. Sloviter, Jason DeBruyne, Roger P. Simon, Robert Meller

**Affiliations:** 1 Department of Neurology, Emory University, Atlanta, GA USA; 2 Morehouse School of Medicine, Neuroscience Institute, 720 Westview Drive SW, Atlanta, GA 30310 USA; 3 Morehouse School of Medicine, Institute of Translational Genomic Medicine, Atlanta, GA USA

**Keywords:** PNES, RNA-Seq, Seizure, Biomarker, Blood, Human, Transient loss of consciousness

## Abstract

**Objectives:**

The ability to differentiate epileptic- and non-epileptic events is challenging due to a lack of reliable molecular seizure biomarker that provide a retrospective diagnosis. Here, we use next generation sequencing methods on whole blood samples to identify changes in RNA expression following seizures.

**Methods:**

Blood samples were obtained from 32 patients undergoing video electroencephalogram (vEEG) monitoring. Blood samples were collected in PaxGene tubes at baseline (admission) and following a seizure event (4–6 h and 24 h later or discharge). EEG and video of clinical events were reviewed by the clinical team and study epileptologist and were classified as epileptic seizure, psychogenic nonepileptic spell (PNES), or other. RNA was extracted from blood and RNA expression was determined using RNA-sequencing.

**Results:**

We show significant differences in RNA profiles between patients that did or did not experience an epileptic seizure. Compared to baseline patients with PNES show large increases in RNA expression 4–6 h and 24 h post seizure. Conversely, genes that changed following epileptic seizure showed more modest changes associated with a decrease in immune system function. Transcript usage was changed between patients with PNES and epileptic seizure at all three time points examined. Lists of genes differentially expressed following PNES or epileptic seizure vs. all baseline samples were used as classifiers for prediction. Models generated using random forest and radial support vector machine algorithms were 100% accurate at predicting both PNES and epileptic seizures.

**Significance:**

These data suggest that blood gene expression changes may have utility to retrospectively identify patients who have suffered a seizure or seizure-like event as a cause of transient loss of consciousness.

**Supplementary Information:**

The online version contains supplementary material available at 10.1007/s00415-024-12877-1.

## Introduction

Epilepsy affects more than 3 million people worldwide and is characterized by repeated unprovoked seizures associated with many causes. The differential diagnosis of seizures is based on patient history and clinical examination, and misdiagnosis is frequent [1, 2]. It is often challenging to determine retrospectively whether a patient has suffered a seizure or a non-seizure event. Of patients who report a previous seizure in an emergency room setting, 10–20% may have suffered a psychogenic non-epileptic spell (PNES). Video electro-encephalogram (vEEG) is the gold standard for diagnosis; however, it is only performed in specialized epilepsy centers prospectively, and has poor retrospective diagnostic accuracy.

Psychogenic non-epileptic spells (PNES) are recognized as a conversion disorder (DSM-V). Typically, a patient with PNES displays the behavioral signs of a seizure but does not have ictal epileptiform activity or post-ictal memory loss and confusion [3]. Although vEEG monitoring might clarify diagnosis, vEEG is costly and not easily available outside of epilepsy centers. The misdiagnosis of PNES is a concern for two key reasons. First, while PNES is responsive to therapy [4], therapy delays (due to diagnostic delays) lead to lower response rates. Indeed, the typical delay for confirmation of PNES is estimated to be 7 years [3], causing considerable costs for both the patient and the health system. Second, delayed diagnosis leads to inappropriate medication with anti-epilepsy drugs (AED). Studies of patients with a vEEG-confirmed PNES diagnosis show that 80% of patients were taking anti-seizure medication [5]. Therefore, a retrospective diagnostic test to distinguish epileptic seizure from PNES is needed.

Here, we describe our studies of blood gene expression changes in patients undergoing long-term vEEG monitoring in an Epilepsy Monitoring Unit. Our data show that post-seizure changes in blood transcriptome profiles may enable the retrospective analysis of seizure-like events.

## Methods

### Ethics approval and patient consent statement

All human studies were reviewed and approved by Morehouse School of Medicine Institutional Review Board (IRB) (Protocol # 1536255 initial approval date 01/14/2020) and acknowledged by the Emory University IRB. All participants or guardians gave written informed consent prior to their enrollment in the study. Samples were de-identified prior to analysis.

### Participant recruitment

Human participants were recruited at the Emory University Hospital Epilepsy Monitoring Unit (EMU) between July 2020 and July 2023. Participants were electively admitted for video electro encephalogram (vEEG) monitoring for clinical purposes including characterization of seizures or seizure-like spells, or for localization of epileptic seizures. Adult patients (age ≥ 18 years) who were seizure free for 5 days prior to admission (self-reported) and who could provide informed consent were recruited. Exclusion criteria included participation in any investigational drug treatment within the previous 90 days, or gene or cellular therapy at any time [6–8]. Consent was obtained by a research coordinator/ EMU staff member prior to initiating EEG monitoring.

All patients underwent vEEG monitoring to record events of interest. Patients were monitored continuously for immediate reporting of events. Event characteristics and EEG findings were documented in the clinical chart at the time of the event by the EMU clinical team (nurse practitioner, epilepsy or clinical neurophysiology fellow, and board-certified epileptologist). EEG and clinical records were reviewed and interpreted by the study epileptologist (KLB) to classify the event type (i.e. epileptic seizure vs. PNES vs. other), classify the seizure type (i.e. focal vs. generalized), record the time of occurrence, and record the duration of the event. For any events that could not be classified immediately, the blood collection procedure was initiated, and diagnosis was confirmed upon later review of EEG. Seizure types were classified according to the ILAE 2017 guidelines [9, 10].

Of 260 admissions, 258 patients were approached. Of these 258 patients, 136 were deemed ineligible due to having had a seizure or seizure-like event within the previous 5 days (an exclusion criterion). 19 patients declined to participate in the study. In all, 77 patients were deemed eligible and consented to participate in the study (30% of those approached). Of these 77 consenting patients, 41 had a seizure-like event (15% of patients approached), 3 withdrew, and 33 left without a seizure occurring (Table 1). Data from 8 patients were lost due to RNA extraction issues (failed QC). 33 patients’ samples were sequenced.

**Table 1:** Patient data

	Baseline	4–6 h	Discharge
**PNES (No EEG seizure)**^a^	**4**	**4**	**3**
*Other*^b^	1	1	1
**Epileptic Seizure (EEG verified)**	**27**	**27**	**24**
**Generalized**	**6**	**6**	**6**
*GTC*	5	5	5
*GIA*^c^	1	1	1
**Focal**	**21**	**21**	**18**
*FTC*	7	8	5
*FIA*	13	12	12
*Sub_clinical*^a^	1	1	1

^a^Diagnosis of PNES made when both no ictal activity is seen on EEG and clinical semiology is consistent with diagnosis of PNES

^b^Diagnosis of not PNES/ other. No EEG activity but clinical semiology consistent with diagnosis of epileptic seizure. This sample was excluded from all analysis

^c^Denotes samples removed from second analysis

### Sample collection

Baseline blood samples (RNA and DNA) were obtained upon admission at the beginning of vEEG monitoring. Once an EEG confirmed epileptic seizure or PNES event was reported, blood samples were obtained 4–6 h after the event, and 24 h post event termination or at discharge. In cases of multiple seizures, we obtained the 6 h blood sample following the first seizure, and the 24-h sample 24 h after the last seizure. All blood samples were stored at Emory prior to transfer (frozen) to Morehouse School of Medicine. DNA samples were banked for future use.

### Data collection

Patient demographics and clinical phenotype were obtained by retrospective chart review. Information collected included age, sex, race, home, anti-seizure medications, serum drug levels, in-hospital administration of anti-convulsive medication (drug, dose and duration), history of epilepsy, prior epilepsy work up including imaging findings, other medical history, and compliance with anti-seizure treatment. Race was accepted as self-described.

### Library assembly

RNA was extracted from blood samples (stored in Paxgene tubes at −20 °C) using the Pre-Analytix RNA extraction kit (Qiagen). RNA-seq libraries were constructed using the Ion Total RNA-Seq Kit v2 (ThermoFisher Scientific) with 500 ng total RNA as starting material (not globin depleted). RNA was sheared, ligated to adapters, and reverse transcribed with Super Script^TM^III. cDNA was size selected using Ampure XL beads (Beckman Coulter) and amplified using Platinum™ DNA polymerase (14 cycles) with IonXpress barcode primers (1:16). Libraries were quantified using High Sensitivity DNA Bioanalyzer chips, pooled and cloned onto sequencing spheres, loaded on Ion 540 chips, and sequenced (Ion Torrent S5).

### Alignment of sequencing data to the reference genome

Human data were processed on a Dell R7525 server running Ubuntu 20.04. Unaligned Binary alignment matrix (Bam) files were written to fastq, trimmed and then aligned with STAR (2-pass mode)/ Bowtie2 to the main chromosomes of the GRCh38 reference genome (full scripts available at https://github.com/rob-meller). Resultant Bam data files were analyzed in Partek Genome Studio software (v7) using the Ensembl v109 annotation guide for GRCh38 or used for transcript identification in stringtie2.

### Differential expression and transcript analysis

Gene expression was calculated as reads per kilobase of transcript per million mapped reads (RPKM) using Partek Genomic Suite (V 7.0). Genes with fewer than 10 aligned reads in 50% of samples were filtered out. The rpkm values were normalized by dividing by the trimmed mean. Data were subjected to multifactorial analysis of variance (ANOVA or ANCOVA) and RNAs with a 1.5-fold difference in expression levels with significance set at *p* < 0.05 (FDR corrected p value) were considered.

The quantification of both known and novel transcripts was derived from the output GTF file generated using StringTie (v2.2.1), and count matrix was assembled using a custom python script (PrepDE.py). Statistical analysis was performed using the edgeR-limma framework in R (v4.3). To control random effects, a sample ID was incorporated into the model. The log2 fold-change (log2FC) values were computed for each comparison, and expression was subjected to Bayesian smoothing of standard errors. Differential isoform expression was determined by log2FC and a false discovery rate (FDR) corrected p-value below 0.05. Transcript isoform switching or differential transcript usage (DTU) analysis was performed using IsoformSwitchAnalyzeR package in R [11]. We set a differential isoform fraction (dIF) cutoff = 0.1 to identify significant changes and excluded genes with only one isoform. Splicing patterns and their functional consequences were determined using extractSplicingSummary and extractConsequenceSummary functions.

### Prediction modeling

The TMM gene expression data matrix was loaded into R as a csv file, and expression values filtered using lists of expressed genes between baseline and either 4–6 h Seizure samples, or 4–6 h PNES samples. The R package Caret [12] and CaretEnsemble were used to run prediction modeling. A copy of the script is in our repository.

### Data availability

Sequencing data and clinical phenotype data are available at dbGAP** (**study phs003460.v1.p1). Summary gene expression data is provided in the supplementary data file, along with ANOVA result tables and Gene ontology tables. These data and copies of scripts are also available on the GitHub page (https://github.com/rob-meller) or contact rmeller@msm.edu.

## Results

### Time course of differential gene expression following seizure events.

We investigated the blood transcriptome from patients undergoing vEEG monitoring in an epilepsy monitoring unit. We sequenced 33 sets of patient samples from our cohort (91 samples total), yielding on average 19 M ± 9.5 M reads/sample. Of the RNA-derived reads, 9.2% ± 2.9 aligned to exons, 7.7 ± 1.6 aligned to partial exonic regions, 73 ± 3.1% aligned to introns, and 9.8 ± 1.2% aligned to intergenic regions. We removed one set of patient samples as they only had one viable timepoint RNA-seq data, and one patient was excluded with an “other” seizure classification (see Table 1).

**Fig. 1:**
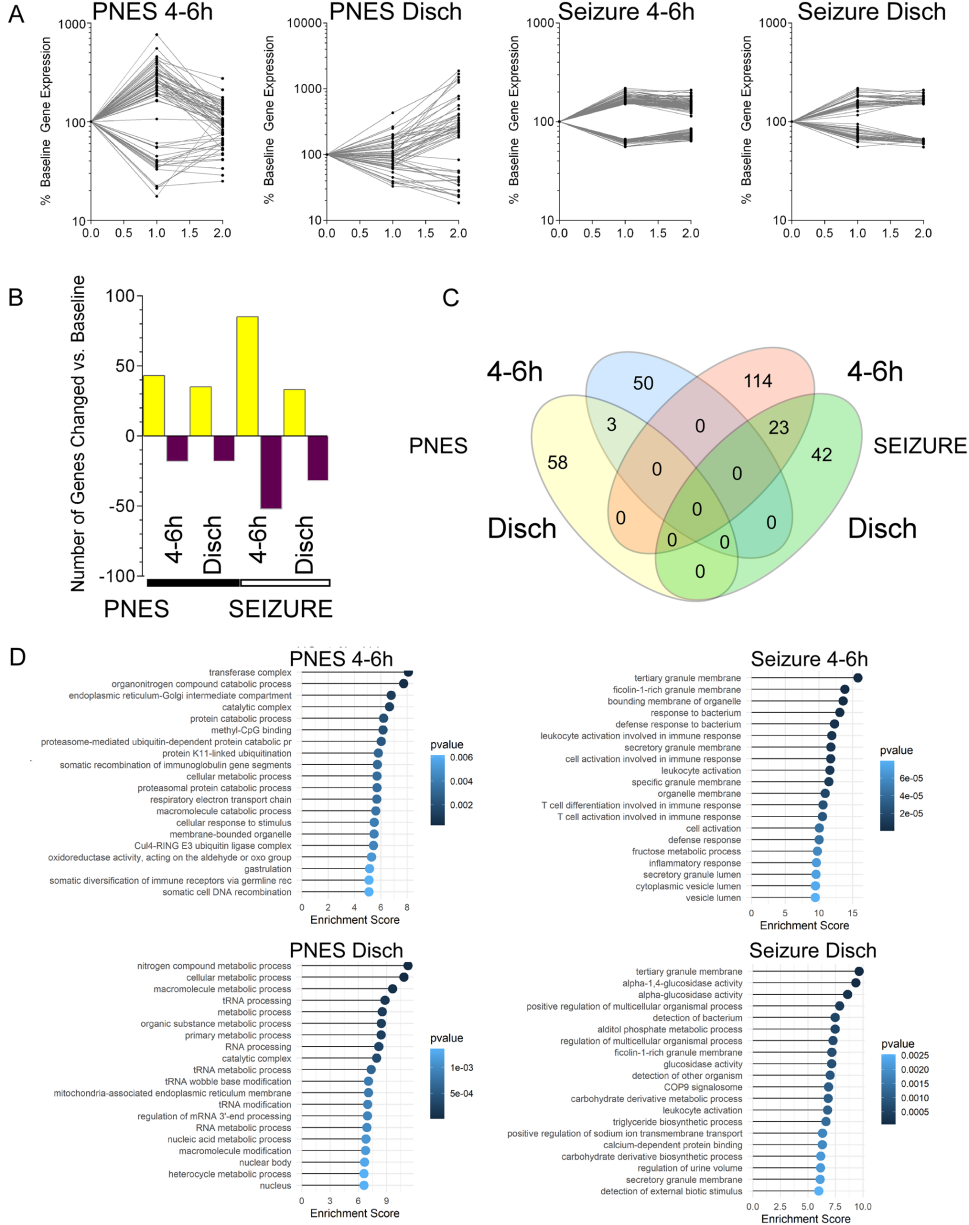
Time course of differential gene expression following EEG-verified seizure. **A** Line graph of the mean gene expression of DEGS, normalized to the baseline value (100%). Data are separated based on the gene lists of DEGS for each condition relative to the baseline samples. **B** Bar chart of number of DEGs identified following 3-way ANOVA (factors: seizure * time interaction, sampleID; p < 0.05 (FDR adjusted) 1.5-fold change) (contrasts: seizure * time interaction vs. baseline). The number of genes increase is represented in yellow and decrease in dark blue. **C** Venn diagram showing overlap of differentially expressed gene identifier names in various groups vs relevant baseline samples. Note the lack of overlap between the seizure data and the PNES data, suggesting different sets of genes show differential expression over time. **D** Gene ontology analysis of DEGs relative to baseline. Enrichment scores and p values of top 20 pathways represented by lollipop plot

We first investigated gene expression over time with respect to baseline samples for patients with EEG-confirmed seizure, or patients with no EEG seizure activity/PNES. We observed 137 genes differentially expressed between baseline and 4–6 h post event in the seizure group (85 increased/52 decreased) vs. 61 in the PNES group (43 increased/18 decreased), and 65 genes were differentially expressed between the baseline and discharge sample in the seizure group (33 increased/32 decreased) vs. 53 genes in the PNES group (35 increased/18 decreased) (Fig. 1A). When the mean of each gene expression change was plotted with respect to baseline the PNES group genes showed larger changes compared to the seizure group (Fig. 1B). Interestingly, there was no overlap of genes between the seizure and PNES groups (Fig. 1C).

### Gene ontology analysis of time course analysis

Pathway analysis was performed on differentially expressed genes using the Partek Genomics Studio GO enrichment tool (see Supplementary Data Table 2), and the top 20 pathways were reported. In the PNES group, there is an enrichment of protein catabolism-associated pathways, especially ubiquitin proteasome system-mediated protein degradation (Fig. 1D). At discharge, the PNES group pathways converge on RNA processing and metabolism. In contrast following an EEG-verified seizure, pathway analysis showed an enrichment of immune system pathways, which were also enriched at discharge (Fig. 1D). Interestingly, when we split the DEGs for increased and decreased expression, the decreased expressed genes mapped to these immune system pathways, whereas increased DEGs showed less enrichment (not shown). This suggests that the strongest biological response to an EEG-verified seizure is depression of immune system pathways.

### Blood transcriptome response to an EEG-confirmed seizure

**Fig. 2:**
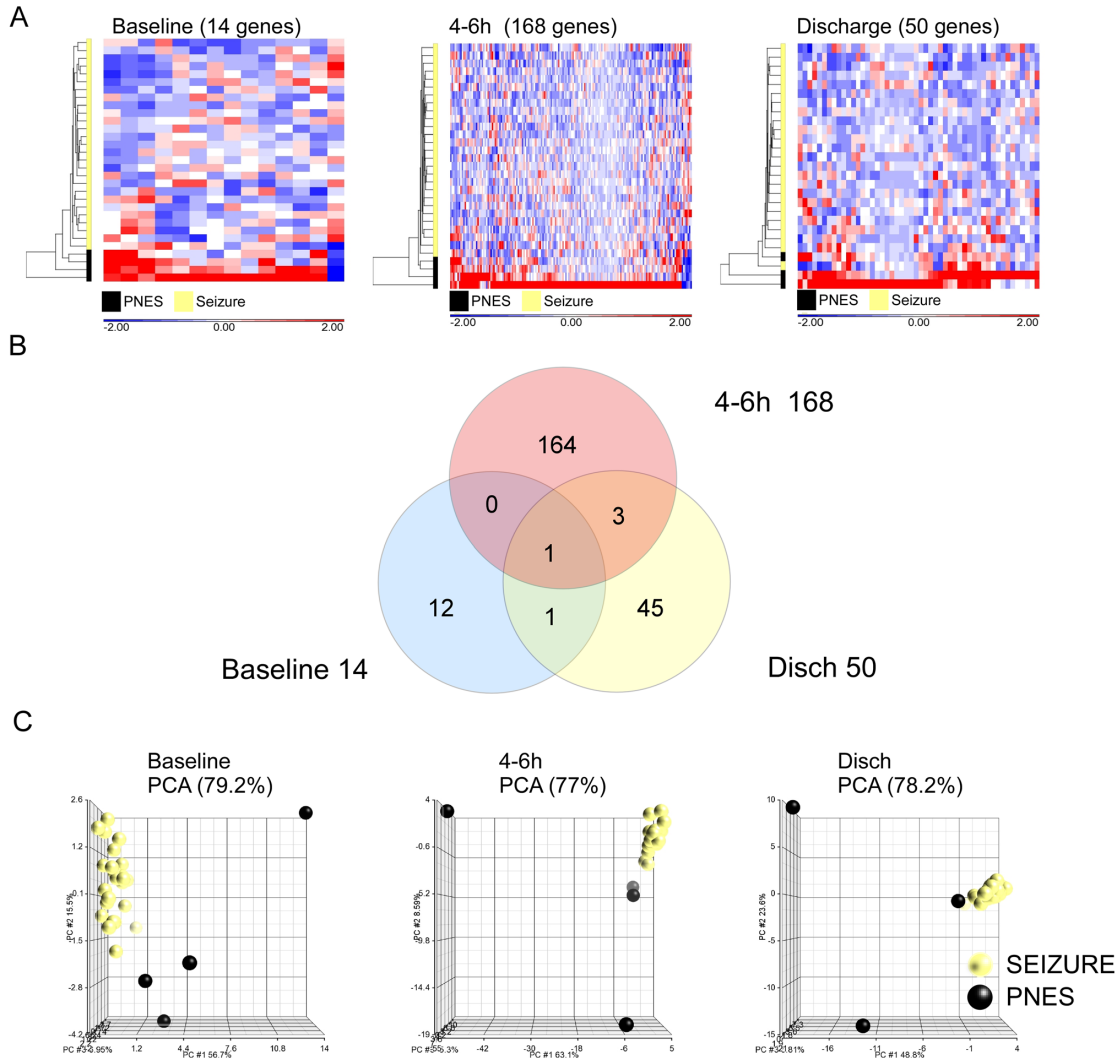
Differential gene expression between EEG-verified seizures and seizures without EEG changes. Hierarchical clustering of gene expression values different between attributes (seizure/PNES) at various time points following a seizure event. Data were subjected to 5-way ANCOVA (factors: race, sex, age, seizure * time interaction, sample-ID) (*p* < 0.05) to create the gene expression matrix of 14, 168, and 50 at baseline, 4–6 h, and discharge, respectively. At each time point, DEGs were clustered by sample and gene expression using Euclidean distances (average linkage). **B** Venn diagram showing overlap of differentially expressed gene identifier names in each time group analysis (PNES vs seizure). Note the lack of overlap. **C** Principal component analysis of DEG data matrix at each time point shows separation of PNES/ Seizure data groups. The three principal components account for 79.2%, 77% and 78.2% of variation at baseline, 4–6 h and discharge, respectively

We then investigated the gene expression response between PNES and EEG-verified seizure groups at different times post seizure (baseline, 4–6 h, and discharge). In Baseline samples, 14 genes showed 1.5-fold change (*p* < 0.05 FDR adjusted) (Fig. 2A). There were more genes differentially expressed between seizure and PNES groups at both 4–6 h (168) and at discharge (50) compared to baseline (± 1.5-fold change, FDR adjusted *p* < 0.05) (Fig. 2A). The DEGs were subjected to hierarchical clustering which showed PNES group patients had large numbers of genes increased compared to Seizure group patients at each time point.

Few genes showed differences between seizure and PNES groups at all three time points (1) (Fig. 2B). PCA of the differentially expressed genes between seizure and PNES groups (Fig. 2C) showed a separation of the PNES patients compared to the seizure group. PCA showed that three components account for approx. 79.2% of variance at Baseline, (first three components were 56.7, 15.5, 4.0, respectively), 77% at 4–6 h post seizure (first three components were 53.1, 8.6, 3.8, respectively), and 78.2% at discharge (first three components were 48.8, 26.6, 3.8, respectively) (Fig. 2C).

### Alternative transcript usage following seizures

We first compared transcript usage between post-seizure groups and baseline. We observed that 52 transcripts were expressed at 4–6 h post-event in the PNES group, but only nine in the post seizure samples. There were no transcripts differentially expressed between baseline and discharge samples for either PNES or seizure groups.

**Fig. 3:**
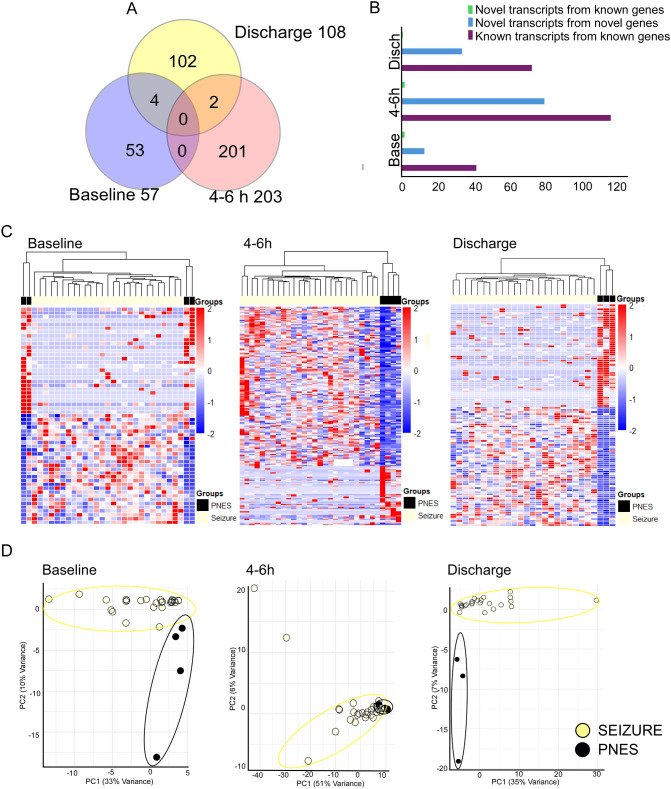
Differential transcript expression (DTE) analysis in PNES and seizure across time points. **A** Unique differentially expressed transcripts specifically identified at 4–6 h post-seizure (201) and at the discharge time point (102). **B** The distribution of novel transcripts compared to known transcripts showed a significant contribution across all time points. **C** Hierarchical clustering of transcript expression values between PNES and seizure at different time points identified 57, 203, and 102 transcripts at baseline, 4–6 h, and discharge, respectively [FDR-corrected *p*-value < 0.05; 1.5-fold change]. **D** Principal component analysis of DTE shows distinct separation between conditions. The three principal components observed 43%, 57% and 42% variance at baseline, 4–6 h and discharge, respectively

We found 57, 203, and 108 differential transcript events between PNES and Seizure at baseline, 4–6 h post-seizure, and discharge, respectively (Fig. 3A). Of these events, there were few transcripts identified as differentially expressed at multiple time points between Seizure and PNES (Fig. 3A, C). The software determined most transcripts were previously known in baseline, 4–6 h, and discharge samples (42,117,73, respectively), but a small number of novel transcripts were identified for novel genes in baseline, 4–6 h, and discharge samples (13;80,34, respectively) and novel transcripts from known genes (2, 2, 1, respectively) (Fig. 3B). Notably, we observed that novel transcripts were more downregulated in baseline and discharge samples but upregulated in 4–6 h samples while most known transcripts followed upregulation on comparison between PNES to seizure samples (Fig. 3B). PCA analysis revealed a high variance of 57% in the 4–6 h timeframe. A distinct separation between conditions was also discernible in the PCA plots of the baseline and discharge samples, where variances of 43% and 42% were observed, respectively (Fig. 3D).

### Differential transcript usage (DTU), and functional consequences of isoform switching,

**Fig. 4:**
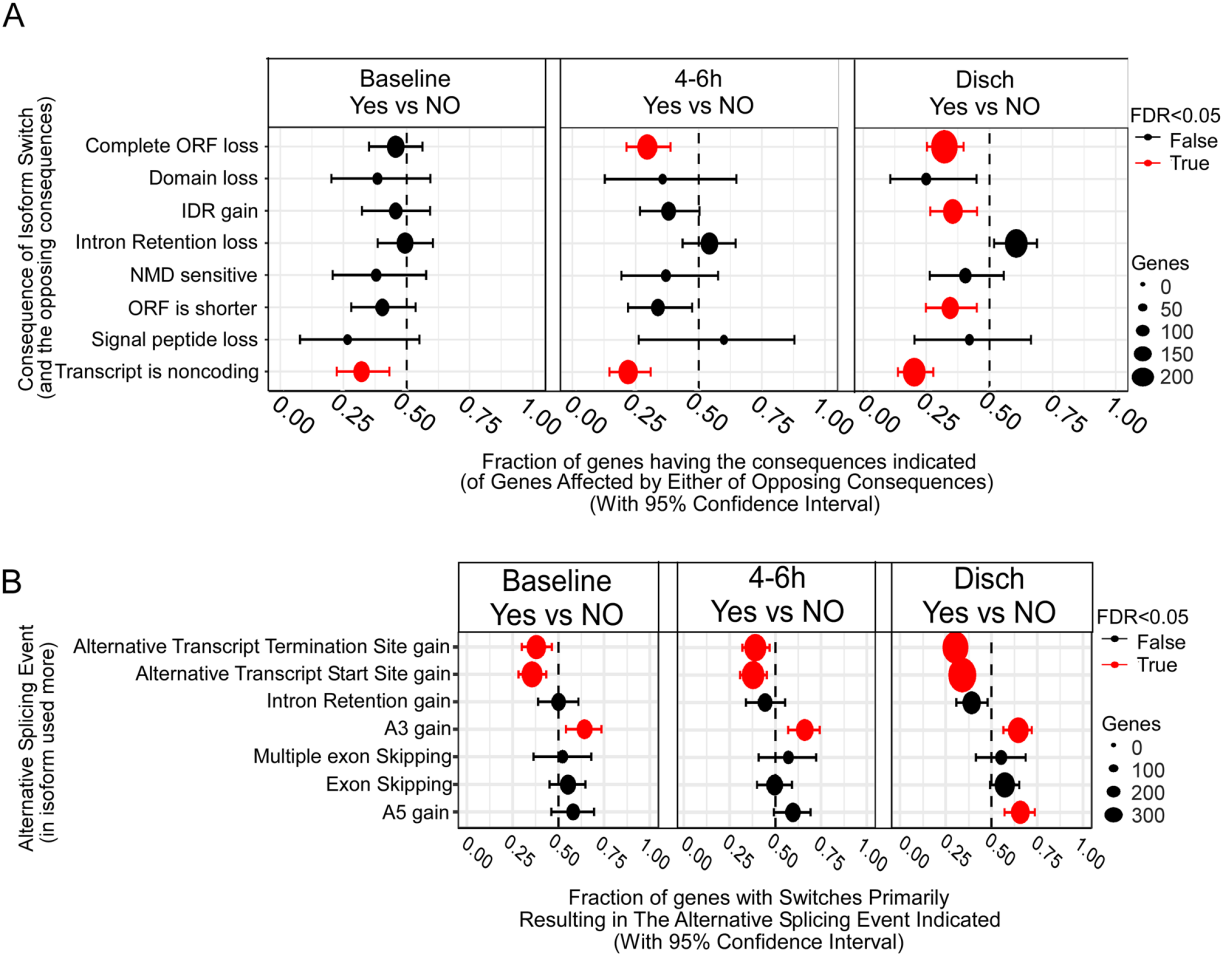
Functional consequences, influence of splicing and top ranked genes with isoform switching in PNES vs Seizure samples. **A** Enriched alterations primarily included amino acid sequence changes like complete ORF loss, domain loss, IDR loss, and transition from coding to noncoding transcripts. **B** Top two ranked genes based on q values for isoform switching between PNES and Seizure groups across baseline, discharge, and 4–6 h post-seizure samples. Identified genes include HLA-E (*q* value = 7.91E^−30^) and ARF3 (*q* value = 2.48E^−20^); LAPTM5 (*q* value = 3.82E^−23^) and PLIN3 (*q* value = 9.21E^−21^); USP7 (*q* value = 3.68E^−15^) and SNRNP200 (*q* value = 6.14E^−13^). **C** Enrichment analysis of splicing events highlighting predominant alternative splicing-related events including aTTS gain, aTSS gain, and alternative 3′ or 5′ splice sites

To understand the underlying mechanism behind a differentially expressed transcript, we calculated transcript usage and found 1518 isoforms linked to 1780 switching events in 931 unique genes across all comparisons (Supplemental Data Table 2). DTU genes were most different between the PNES discharge group and Seizure discharge group (367, see supplemental data), and PNES baseline and Seizure baseline group (41, see Supplemental Data Table 2). The alterations primarily pertained to amino acid sequence alterations due to open reading frame (ORF) loss, domain loss, intrinsically disordered regions (IDR) loss, and the transition of coding transcripts into noncoding transcripts (Fig. 4A). Remarkably, no discernible functional consequences of transcript alterations were observed within the PNES and seizure samples when compared with baseline. For further insight, we focused on the top two ranked genes based on q values of isoform switching in PNES vs. seizure paired at baseline, 4–6 h and discharge. We identified HLA-E (*q* value = 7.91E^−30^) and ARF3 (*q* value = 2.48E^−20^); LAPTM5 (*q* value = 3.82E^−23^) and PLIN3 (*q* value = 9.21E^−21^); USP7 (*q* value = 3.68E^−15^) and SNRNP200 (*q* value = 6.14E^−13^) as top two ranked genes involved in isoform switching across baseline, discharge and 4–6 h post-seizure samples in comparison of PNES vs. Seizure groups, respectively (Supplementary Fig. 1).

To elucidate the underlying mechanism accountable for isoform switches, we conducted an analysis of the occurrence rate of splicing events associated with the detected isoform switches in PNES compared to seizures. We systematically examined the influence of each process on isoform switching across all time points. Our investigation revealed that a combination of alternative splicing (AS), alternative transcription starts site (aTSS), and alternative transcription termination site (aTTS) could collectively influence the alteration of transcript isoform switching. Specifically, we observed that 33.67% (455 genes) of isoform switching events could be attributed to the interaction among these three mechanisms (Fig. 4B). Furthermore, through enrichment analysis of splicing events, we identified aTTS gain, aTSS gain, and alternative 3′ or 5′ splice sites as the predominant alternative splicing-related events (Fig. 4B). These findings suggest that alterations in alternative splicing may contribute to the ability to distinguish between PNES and seizure patients.

### ML-based prediction modeling correctly assigns PNES or epileptic seizure

**Fig. 5:**
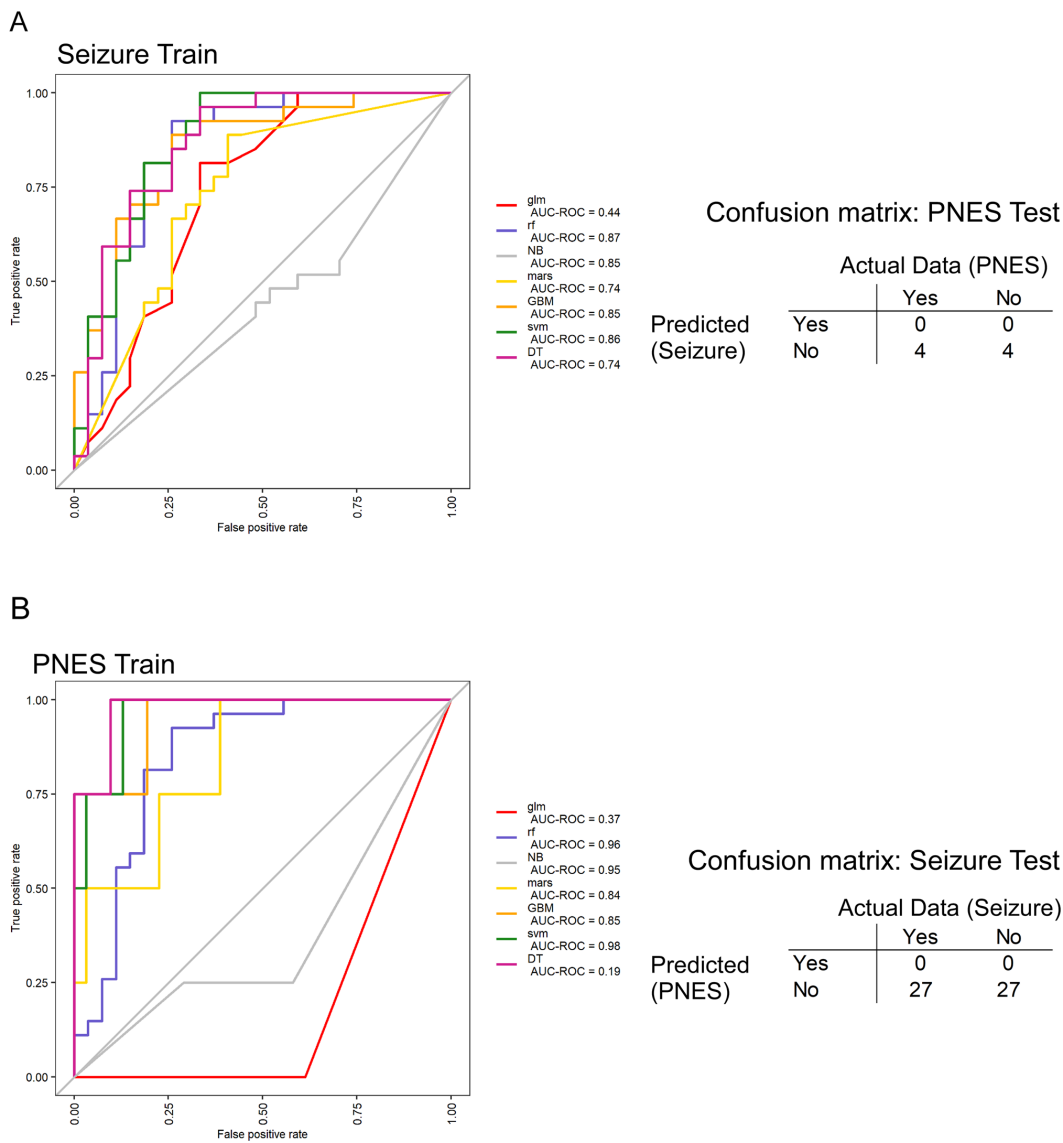
ML-based prediction modeling to distinguish between baseline samples and PNES/ seizure. **A** Combined ROC analysis of 7 prediction modeling algorithms. Data were trained on whether the sample was baseline (*N*) or following a seizure spell (Yes) as a binomial classifier. AUC results are the mean accuracy of tenfold resampling. Confusion matrix shows the results of testing a model to identify seizure in PNES patients. No PNES patients were called as having experienced an epileptic seizure. **B** Combined ROC modeling of models to identify the most accurate models to predict a PNES diagnosis. AUC results are the mean AUC following tenfold resampling. The confusion matrix shows the results of testing the PNES prediction model on seizure patient data. No patient with epileptic seizure, either before of following their clinical event, were classified as having had a PNES event (model abbreviations: glm generalized linear model, rf random Forest, NB naïve Bayes, mars multivariate adaptive regression splines, GBM Gradient boosting machine, svm radial support vector machine, DT decision tree)

Classifier lists of genes were first established following differential gene expression to identify epileptic seizure/PNES specific expression (4-way ANCOVA: controlling for race, age, and sex) and contrasting between all baseline samples and 4–6 h seizure samples (PNES train) or PNES samples (PNES train). We used the top 60 most significant genes from each analysis. We first trained the seizure data (seizure train) using 6 algorithms in the caret package (Fig. 5A). The model was trained on baseline vs. 4–6 h data from patients who had an epileptic seizure (27 patients, 2 samples each), and subjected each to ten-fold cross validation. Random forest, naïve Bayes, radial SVM, and GBM models performed best in training (ROC of 0.87–0.85) (Fig. 5A). These models were then tested on the baseline and 4–6 h PNES data. The models called all samples as no seizure (*N*) (see confusion matrix Fig. 5A). This means a seizure model did not call the PNES data as a seizure. The models tested on the training data were 100% accurate 27 baseline (*N*) samples and 27 seizure (*Y*) samples.

A second model was then trained on the PNES data using the same algorithms (PNES train). Based on ROC analysis, the best models were random forest, radial SVM, naive Bayes and GBM (ROC 0.98–0.85) (Fig. 5B). These models were then tested on the seizure data. The models called all samples (27 baseline and 27 4–6 h post seizure) as no PNES (*N*). This means a PNES model correctly identifies a seizure as not PNES. The models tested on the training data were 100% accurate 4 baseline (*N*) samples and 4 PNES (*y*) samples.

## Discussion

We previously utilized whole transcriptome profiling to identify RNA signatures in whole blood following brain injury [8, 13]. The brain lymphatic system contains lymphocytes that are subjected to the same environmental stress of an acute neurological insult thereby acting as a sentinel [14–18]. Brain endothelial cells change following seizures causing blood brain barrier dysfunction [19], allowing diffusion of factors from the brain or immune cell access to the brain. Our study describes gene expression differences between baseline and following epileptic seizures or PNES. Together these data suggest that blood transcriptome analysis may enable the retrospective diagnosis of seizures.

We noticed different transcriptome responses in patients with psychogenic nonepileptic seizures, compared to epileptic seizures, which could be the basis of a biomarker. Many previous studies have focused on gene expression changes in brain following modeled seizure in animals, whereas few have investigated animal blood [20–22] or human blood. Here, we have focused on blood expression to determine whether such biomarkers may enable a more rapid diagnosis of a seizure having occurred in a patient, versus gaining insight into mechanisms of disease. The pathways we report are more likely reflective of the source of material (immune system/blood vs. other tissues), although inflammatory factor signaling has been shown following seizures [23–25].

Previous blood-based biomarkers of seizures have focused on diagnosing patients with epilepsy, (temporal lobe epilepsy) as a disease state vs. identifying a clinical seizure. Multiple plasma proteins have been suggested to be epilepsy biomarkers, but many are neuronal proteins associated with non-specific brain injury (tau, GFAP, UCHL1, etc.). Prolactin has some utility for determining whether a patient has a seizure, if measured within 1 h of the event, but since other conditions may cause high prolactin levels, this biomarker is not widely used in the ER [26–28]. A recent study suggests that IL6 is released following a seizure but not psychogenic seizures, which may have utility for retrospective seizure detection [25]. Interestingly IL6 affects gene expression in blood [29]. Hence, the signatures we observe may be due to seizure-associated immune signaling changes. This is supported by GO analysis revealing an enrichment of immune system pathways following seizures (Fig. 5). Recent studies show fMRI signal changes in brain following PNES, supporting our data showing a biochemical effect of PNES [30]. In patients who have a mixed disorder including both epilepsy and psychogenic nonepileptic spells, a retrospective biomarker capable of differentiating epileptic seizure from psychogenic nonepileptic spells may also have additional relevance in classifying single events.

There are few NGS gene expression studies in blood following seizures. However, circulating small RNAs may be useful biomarkers of seizures or epilepsy. Specifically, tRNAs appear higher in patients with TLE pre-seizure, than in controls or following a seizure [31], but not in patients with PNES. microRNAs (miRNA) are short (18-mer) RNAs with potential differential levels in plasma and brain in TLE patients [32–35]. We detected both mature miRNA and tRNA in our data (see supplementary data) but did not observe any significant changes even when subsetting data to those biotypes, however most studies use plasma or serum not whole blood.

Gene expression changes have been reported in response to circadian rhythms in blood and other organs [36–40]. Debski in 2020 showed that diurnal brain (hippocampal) transcriptomes are different in control and epileptic mice (evoked using pilocarpine) [41]. However, the effect of response to a seizure was not assessed [41]. Our samples were sampled at all times across the day as seizures occurred at all times of day, and do not show a diurnal bias (Supplementary Fig. 2A). A day/night diurnal pattern on blood gene expression was observed in MS relapse patients [42]. When we perform a similar day-night (AM/PM) analysis of seizure time on our data the impact of time was weak [1.05 mean F ratio vs. 2.1 mean F ratio, PNES vs. epileptic seizure respectively (Supplementary Fig. 2B)]. Similarly, if time was used as continuous factor to correct the data, the effect of time on the variance of gene expression values was much smaller than the variance due to PNES vs. epileptic seizures (Supplementary Fig. 2C). PCA patterns show separation of PNES and epileptic seizure based on corrected DEG lists. Therefore, it is unlikely that the time of day of the clinical event is affecting the differential gene expression we are observing between PNES and epileptic seizures.

### Limitations

Seizures cannot be accurately predicted, hence the blood samples were collected approximately 4–6 h following the event to allow for any necessary acute treatment to occur, events to be confirmed by the clinical team, and phlebotomy to be deployed. We would prefer additional time points to map the temporal transcriptome trajectory following a seizure. In addition, we used a patient as their own control for some analysis, and these may differ from baseline of patients experiencing other forms of temporary loss of consciousness, so would require further testing. Patients entering the epilepsy monitoring unit are doing so for clinical purposes. We lack any ability to control timing of seizure (and thus timing of blood draws), number of seizures, antiseizure medications (ASMs) administered, rescue medications given or discharge timing. Variability existed in the number or types of ASMs given, and some patients had multiple seizures, but the study did not have sufficient power to analyze these factors. The limited sample size was due to study limitations (being performed throughout the COVID pandemic) Where possible, our next goals are to enhance sample size, replicate findings, and test the prediction models on an independent dataset. Finally, while the use of sequencing may currently limit the utility of this approach; our goal was to first show the presence of a blood RNA signature, that may have diagnostic use. If a few signature genes are accurate to perform a predictive test, these could be tested by more rapid PCR based approaches.

## Supplementary Information

Below is the link to the electronic supplementary material.

Supplementary file1 (TIF 1455 KB)

Supplementary file2 (TIF 948 KB)

Supplementary file3 (DOCX 14 KB)

Supplementary file4 (DOCX 15 KB)

Supplementary file5 (DOCX 15 KB)

Supplementary file6 (XLSX 14608 KB)

## Data Availability

All data are available, see Methods.
