# Supplementary figures and images for: Retrospective discrimination of PNES and epileptic seizure types using blood RNA signatures
Source: J Neurol. 2025 Jan 15;272(2):128. doi: 10.1007/s00415-024-12877-1 (PMC11735489; doi:10.1007/s00415-024-12877-1)

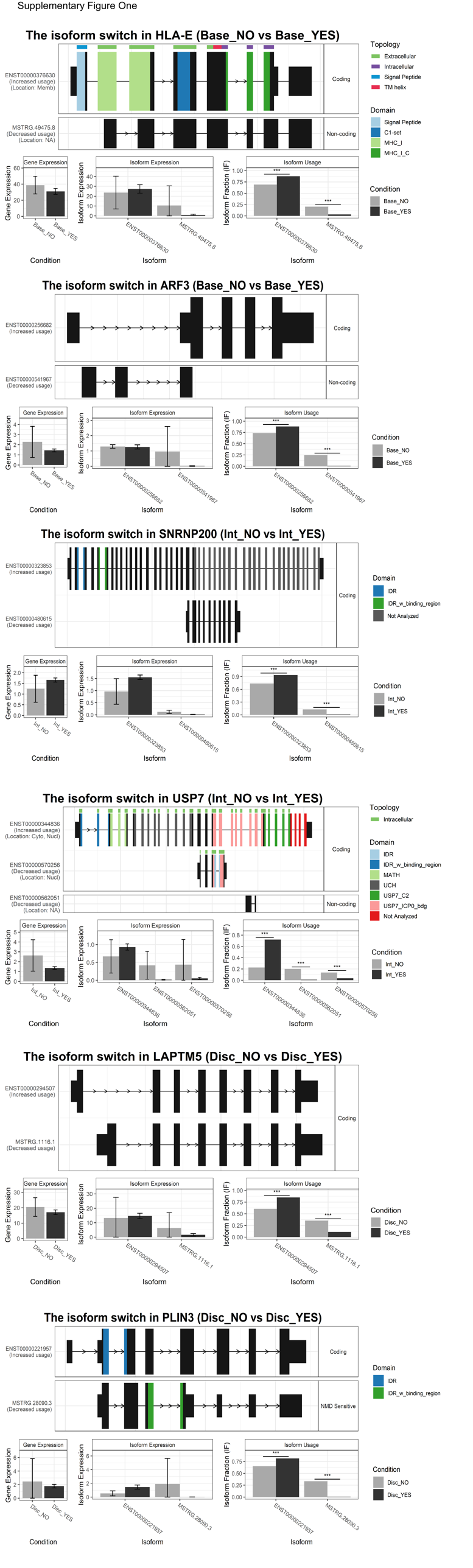

Supplement: Supplementary file 1 — Supplementary file1 (TIF 1455 KB) [file 415_2024_12877_MOESM1_ESM.tif]

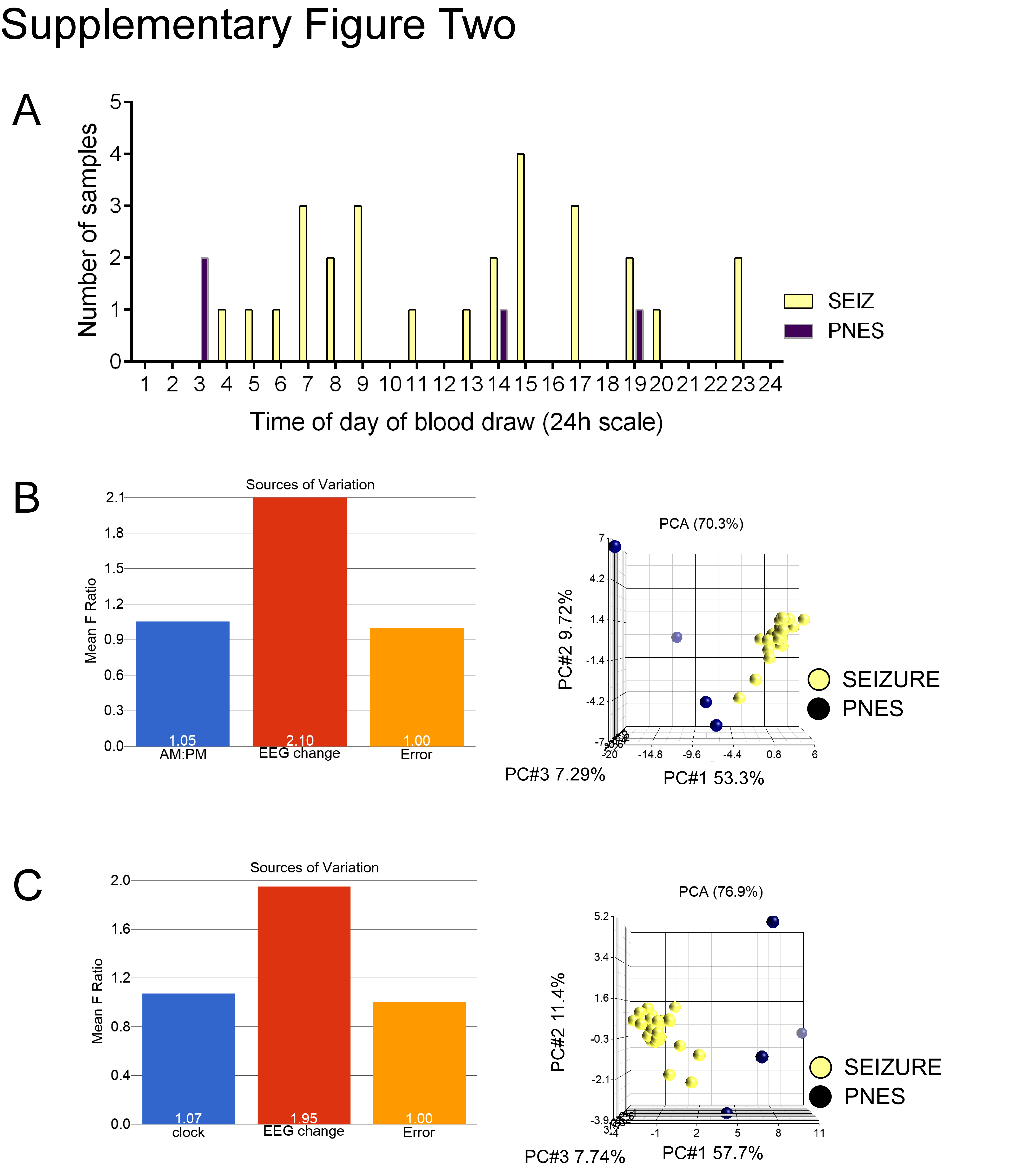

Supplement: Supplementary file 2 — Supplementary file2 (TIF 948 KB) [file 415_2024_12877_MOESM2_ESM.tif]
