# Supplementary material for: Retrospective discrimination of PNES and epileptic seizure types using blood RNA signatures
Source: J Neurol. 2025 Jan 15;272(2):128. doi: 10.1007/s00415-024-12877-1 (PMC11735489; doi:10.1007/s00415-024-12877-1)
Supplement: Supplementary file 3 — Supplementary file3 (DOCX 14 KB) [file 415_2024_12877_MOESM3_ESM.docx]

**Supplementary Figure Legends**

**Fig 1** Functional consequences, influence of splicing and top ranked genes with isoform switching in PNES vs Seizure samples. Examples of differential transcript usage in baseline, 4-6h post seizure and at discharge. Identified genes include HLA-E (q value = 7.91E^-30^) and ARF3 (q value = 2.48E^-20^); LAPTM5 (q value = 3.82E^-23^) and PLIN3 (q value = 9.21E^-21^); USP7 (q value = 3.68E^-15^) and SNRNP200 (q value = 6.14E^-13^).

**Fig 2** Effect of time-of-day correction on seizure patterns of gene expression. A. Histogram of epileptic and PNES seizures showing time of day (24h scale) blood samples were collected for the 4-6h samples. B. Source of variation based on mean F ratio when EEG change and whether the seizure occurred in the AM or PM (categorical Factor). Principal component analysis of DEGS following correction for time of seizure. C. Source of variation based on mean F ratio when EEG change and whether the time of the seizure in 24h scale (continuous variable). Principal component analysis of DEGS following correction for time of seizure.
