# Supplementary material for: Retrospective discrimination of PNES and epileptic seizure types using blood RNA signatures
Source: J Neurol. 2025 Jan 15;272(2):128. doi: 10.1007/s00415-024-12877-1 (PMC11735489; doi:10.1007/s00415-024-12877-1)
Supplement: Supplementary file 4 — Supplementary file4 (DOCX 15 KB) [file 415_2024_12877_MOESM4_ESM.docx]

Supplementary table 1. Correction of DEG list accounting for race age and sex of patients.

*Model Y_ijk_ = µ + Time * change + E _ijk_*

Lm (3-way ANOVA)- Contracts Fishers least significant difference (Time * EEG change)

Compare YES vs NO at each time point.

**Condition # genes Fold change FDR Increased in No Increased in Yes**

Base 22 ± 1.5 p<0.05 20 2

4-6h 308 ± 1.5 p<0.05 259 49

24h 71 ± 1.5 p<0.05 70 1

*Model Y_ijk_ = µ + Time * EEG change + Sample_ID + E _ijk_*

Lm (3-way ANOVA)- Contracts Fishers least significant difference (Time * EEG change)

Compare YES vs NO at each time point.

**Condition # genes Fold change FDR Increased in No Increased in Yes**

Base 8 ± 1.5 p<0.05 8 0

4-6h 271 ± 1.5 p<0.05 233 38

24h 64 ± 1.5 p<0.05 64 0

*Model Y_ijk_ = µ + Time * EEG change + Sample_ID + RACE + SEX + Age + E _ijk_*

Lm (5-way ANCOVA)- Contracts Fishers least significant difference (Time * EEG change)

Compare YES vs NO at each time point.

**Condition # genes Fold change FDR Increased in No Increased in Yes**

Base 14 ± 1.5 p<0.05 13 1

4-6h 168 ± 1.5 p<0.05 161 7

24h 50 ± 1.5 p<0.05 50 0

Here we show the effect of adding various factors to correct for Race and sex of the patient, and its effect on the number of DEGs we identify. We first performed a 2-way ANOVA, looking at the time * EEG seizure group interaction, then added additional factors to control for age race and sex (Table2). In Baseline samples there were 22 genes showing 1.5 fold change (p<0.05 FDR adjusted). There were more genes differentially expressed at both 4-6h (308) and at discharge (71) compared to baseline (+/- 1.5 fold change, p<0.05 FDR adjusted) (Table2). The study has a longitudinal design, so samples across time points may not be independent. We used the sample ID to control random effects / repeated sampling (3-way ANOVA). In Baseline samples there were 8 genes showing 1.5 fold change between YES and NO groups (p<0.05 FDR adjusted), in 4-6h samples 271 genes were different between YES/ NO groups and at discharge 64 genes were different between YES/ NO groups (+/- 1.5 fold change, p<0.05 FDR adjusted) (Table2). Finally, when race, age, and sex of the participants are explicitly controlled for by adding as a factor (5-way analysis of covariance (ANCOVA)) controlled for, the Time * EEG interaction is the largest source of variation (108.95, vs 1.01 (Age),1.48(Race), and 1.45(Sex)). When we extend the model to control for age, sex, and race we observed fewer differentially expressed genes (model reported in the ms). In Baseline samples 14 genes showed 1.5 fold change (p<0.05 FDR adjusted) (Fig 4A). There were more genes differentially expressed between YES and NO groups at both 4-6h (168) and at discharge (50) compared to baseline (+/- 1.5 fold change, p<0.05 FDR adjusted) (Fig 4A). The DEGs were subjected to hierarchical clustering which shows NO group patients had large numbers of genes increased compared to YES group patients at each time point.
