# Supplementary material for: Retrospective discrimination of PNES and epileptic seizure types using blood RNA signatures
Source: J Neurol. 2025 Jan 15;272(2):128. doi: 10.1007/s00415-024-12877-1 (PMC11735489; doi:10.1007/s00415-024-12877-1)
Supplement: Supplementary file 5 — Supplementary file5 (DOCX 15 KB) [file 415_2024_12877_MOESM5_ESM.docx]

Supplemental Table 2. Differential isoform switching following PNES or Epileptic Seizure.

| **The Number of Isoform Switches found were:** | | | | |
| --- | --- | --- | --- | --- |
|  | **Comparison** | **nrIsoforms** | **nrSwitches** | **nrGenes** |
| 1 | Base_PNES vs Base_Seizure | 350 | 304 | 255 |
| 2 | Base_PNES vs Disch_PNES | 223 | 263 | 172 |
| 3 | Base_PNES vs 4-6h_PNES | 371 | 437 | 283 |
| 4 | Base_Seizure vs Disch_Seizure | 58 | 41 | 41 |
| 5 | Base_Seizure vs 4-6h_Seizure | 87 | 58 | 56 |
| 6 | Disch_PNES vs Disch_Seizure | 505 | 503 | 367 |
| 7 | 4-6h_PNES vs 4-6h_Seizure | 357 | 346 | 268 |
| 8 | Combined | 1518 | 1780 | 931 |
